# Lactoferrin promotes hair growth in mice and increases dermal papilla cell proliferation through Erk/Akt and Wnt signaling pathways

**DOI:** 10.1007/s00403-019-01920-1

**Published:** 2019-04-20

**Authors:** Hsiu-Chin Huang, Hsuan Lin, Min-Chuan Huang

**Affiliations:** 1 Renorigin Innovation Institute Co. Ltd., Taipei, Taiwan; 2 Graduate Institute of Anatomy and Cell Biology, College of Medicine, National Taiwan University, No. 1, Sec. 1, Ren’ai Road, Taipei, 100 Taiwan

**Keywords:** Lactoferrin, Alopecia, Hair, Dermal papilla

## Abstract

Hair loss affects men and women of all ages. Dermal papilla (DP) plays a crucial role in regulating the growth and cycling of hair follicles. Lactoferrin (LF) exhibits a wide range of biological functions, including antimicrobial activity and growth regulation. However, its effect on DP and its role in hair growth remain unknown. In this study, we found that bovine LF (bLF) promoted the proliferation of DP cells and enhanced the phosphorylation of Erk and Akt. The bLF-mediated proliferation was significantly blocked by the Erk phosphorylation inhibitor PD98059 or the Akt phosphorylation inhibitor LY294002. Moreover, biotin-labeled bLF could bind to DP cells, and the binding was independent of lipoprotein receptor-related protein 1, a known LF receptor. Importantly, bLF stimulated hair growth in both young and aged mice. Moreover, we also found that bLF significantly induced the expression of Wnt signaling-related proteins, including Wnt3a, Wnt7a, Lef1, and β-catenin. The bLF-mediated DP cell proliferation could be significantly reversed by the Wnt pathway inhibitor XAV939. Our findings suggest that bLF promotes hair growth in mice and stimulates proliferation of DP cells through Erk/Akt and Wnt signaling pathways. This study highlights a great potential of the use of bLF in developing drugs to treat hair loss.

## Introduction

Although hair loss is not a lethal disease, it affects social and psychologic well-being. According to the previous study [32], androgenic alopecia (AGA) affects about 80% of Caucasian men and increases with age. Female pattern hair loss is estimated to occur in 32% of women in the ninth decade of life. Minoxidil and finasteride are the only two AGA treatments approved by the US Food and Drug Administration (FDA). Many new treatments have been introduced in recent years [36]. However, the effects of these treatments are not well characterized. In most cases, the results vary or present with unpredictable side effects. Most of them are trying to convert hair follicles from the telogen to anagen phase.

The adult hair follicle comprises three phases of hair cycle: the growth phase (anagen), the regression phase (catagen), and the resting phase (telogen) [10]. Hair growth cycle is dependent on the interaction of epithelial and specialized mesenchymal cells called dermal papilla (DP), which are located at the base of the follicle. To stimulate the initiation of anagen, DP cells generate instructive signals to induce epithelial bulge cell proliferation [25]. A previous study [4] also indicated that DP cells play an important role in initiating anagen by inducing stem cells. Moreover, hair follicles with a normal keratinocyte compartment fail to generate new hair shafts if the density of DP cells is under the critical threshold.

Lactoferrin (LF; molecular weight, ~ 80 kDa) is an iron-binding glycoprotein belonging to the transferrin family. LF can be found in many exocrine glands, pre-neoplastic and neoplastic lesions [34], and body fluids, including saliva, tears, semen, pancreatic fluids, bile, vaginal secretions, and milk. LF is a pleiotropic protein [21] which exerts effects on antimicrobial activity, immune system modulation, embryonic development, cell growth and differentiation, endothelial cell adhesion, myelopoiesis, cytokine and chemokine regulation, inflammatory response, and immunomodulatory activities [35]. However, its role on hair follicles has received little attention.

The present study addresses the effects of bovine LF (bLF) on hair growth and the underlying mechanisms involved. The results suggest that bLF can act as a growth factor to stimulate DP cell proliferation and hair growth and highlight the potential of LF as a novel agent for alopecia treatment.

## Materials and methods

### Cell culture

DP cells were obtained from rat whisker follicle as previously described [1]. Briefly, 8-week Sprague–Dawley rats (BioLASCO, Taipei, Taiwan) were euthanized by CO_2_, and the whisker pads were removed from rats. The tissues were quickly washed in phosphate-buffered saline (PBS) and then transferred to Dulbecco’s modified Eagle’s medium (DMEM) (Gibco; Thermo Fisher Scientific, Waltham, MA, USA) supplemented with 10% fetal bovine serum (FBS) (Gibco), 100 U/mL penicillin, 100 μg/mL streptomycin, and 0.5 μg/mL fungizone (Gibco). After the removal of the adhering dermal tissues, 20 isolated follicles were transferred to fresh growth medium and cut into small pieces. Cells were incubated at 37 °C in a humidified atmosphere containing 5% CO_2_ in air. DP cells were obtained after 7 days. To confirm the identity of DP cells, the alkaline phosphatase (AP) activity was analyzed using Red-Color AP staining (System Biosciences, Mountain View, CA, USA). We have performed experiments using 5–8 passages of DP cells. LY294002 and PD98059 were purchased from Sigma. XAV939 was purchased from Abcam (Cambridge, UK).

### Cell proliferation assay

Cells were seeded at a density of 2 × 10^3^ cells in 96-well tissue culture plates. After 24 h, the cells were treated with 20–100 μg/mL bLF and incubated for different time periods (1–5 days). After different culture times, cells were incubated for 4 h at 37 °C with 0.5 mg/mL MTT, and then, 10% sodium dodecyl sulfate (SDS) containing 0.01 N HCl was added to dissolve formazan crystals. Finally, the absorbance was measured at 570 nm and 630 nm using VersaMax ELISA microplate reader spectrophotometer (Molecular Devices, Silicon Valley, CA, USA).

### Western blot analysis

Protein lysates of cells were prepared using NP40 lysis buffer. Then, 30 μg of proteins was separated by 8% SDS-PAGE and then transferred to polyvinylidene difluoride (PVDF) membranes. The membranes were incubated with blocking buffer and then incubated with the primary antibodies for pAkt (#4060), Akt (#4691), pErk1/2 (#9101), Erk1/2 (#9102) (Cell Signaling Technology Danvers, MA, USA), Wnt3a (GTX128101), Wnt7a (GTX128106), Lef1 (GTX129186) (GeneTex, Taiwan) and β-actin (Sigma-Aldrich, St Louis, MO, USA), followed by incubating with secondary antibodies peroxidase-AffiniPure goat anti-mouse (115-035-003) or anti-rabbit (111-035-144) antibody (Jackson ImmunoResearch Laboratories, West Grove, PA, USA).

### Biotinylation of LF and cell surface binding assay

bLF was purchased from New Bellus Enterprise Co., Ltd (Tainan, Taiwan). Biotinylation of bLF was performed as previously described [28]. Briefly, bLF was incubated with biotin (Thermo Fisher Scientific) on ice for 2 h. Unlabeled proteins were removed by dialysis against 1 L of ddH_2_O twice. The reaction of bLF binding to DP cells was performed according to a previous report [28]. Briefly, DP cells were seeded at a density of 1 × 10^4^ cells in 96-well tissue culture plates and incubated with 0.1 μΜ biotin-labeled bLF or biotin-labeled BSA in the presence or absence of 100-fold unlabeled 10-μΜ bLF or BSA and recombinant receptor-associated protein (RAP) (Progen, Heidelberg, Germany) at 4 °C. After 4 h, the cells were washed and incubated for 1 h with HRP-conjugated avidin (BioGenex Fremont, CA, USA) at room temperature (RT). The biotin–avidin complex was detected with the *o*-phenylenediamine (OPD) substrate reagent from Sigma-Aldrich (P8787). Reactions were stopped with 3 N HCl and read at 492 nm using an automated plate reader (molecular devices).

### Immunofluorescence microscopy

DP cells were seeded at a density of 1 × 10^5^ cells in 6-well tissue culture plates. After 24 h, the cells were incubated with biotin-labeled bLF or BSA for 1 h and then fixed with 100% methanol. After 5 min, the cells were washed and incubated with PBS containing 10% FBS for 20 min. Then, the blocking buffer was removed, and streptavidin-FITC conjugate (Jackson ImmunoResearch Laboratories) was added; this was followed by incubation in the dark at RT for 1 h, after which the cells were washed and observed under a fluorescence microscope.

### Hair growth in mice

All animal experiments were approved by the Institutional Animal Care and Use Committee of the National Taiwan University College of Medicine, Taipei,Taiwan (IACUC: 20160283)*.* To investigate the influence of bLF on hair growth, 2-month-old and 1-year-old female mice (C57BL/6) (BioLASCO) were anesthetized, and the dorsal hair were removed by a wax–rosin mixture as previously described [30]. After depilation for 1 day, bLF or ddH_2_O control was applied on the back twice daily for 7–12 days. Hair growth was quantified by analyzing the grey-scale of images of the dorsal skin using ImageQuant TL software and normalized to their levels at day 0. *p < 0.05.

### Real-time reverse transcription polymerase chain reaction (RT-PCR) analysis

Total RNA was extracted from DP cells using TRIzol™ Reagent (Invitrogen; Thermo Fisher Scientific), and cDNA was synthesized using reverse transcription kit (Applied Biosystems; Thermo Fisher Scientific). For real-time PCR, QuantStudio 3 Real-Time PCR System (Thermo Fisher Scientific) was used. Total reaction volume of 20 μL contained 1-μL cDNA, 500 nM of each primer, and 10-μL SensiFAST™ SYBR Lo-ROX Kit (Bioline, Taunton, MA, USA). Samples were analyzed in triplicate, and *β-actin* was used as an internal control to normalize the relative quantity of gene expression. For PCR, total volume of 50 μL contained 1-μL cDNA, 200 nM of each primer, Dream Taq DNA Polymerase and Dream Taq buffer (Thermo Fisher Scientific) for 35 cycles. The following primer pairs were used: *Wnt3a*, 5′-AGTGCAAATGCCACGGACTA-3′ (forward), 5′-TTGGGCTCGCAGAAGTTAGG-3′ (reverse); *Wnt7a*, 5′-CAGAATGCCCGAACCCTCAT-3′ (forward), 5′-TAGCCT GAGGGGCTGTCTTA-3′ (reverse); *β-catenin*, 5′-CCATCACCACGCTGCATAAT-3′ (forward), 5′-GAGCAGACAGACAGCACCTT-3′ (reverse); *Lef1*, 5′-GGCATC CCTCATCCAGCAAT-3′ (forward), 5′-GTTGATAGCTGCGCTCTCCT-3′ (reverse); *Gsk3b*, 5′-AGAACCACCTCCTTTGCGGA-3′ (forward), 5′-GTGGTTACCTTGCTGCCATCT-3′ (reverse); *Fzd7*, 5′-GGTGGATGGTGACCTACTCA-3′ (forward), 5′-GCTCGTAAAAGTAGCACGCC-3′ (reverse); and *β-actin*, 5′-AAGATCCTGACCGA GCGTGG-3′ (forward), 5′-CCGCTCATTGCCGATAGTG-3′ (reverse).

### Statistical analyses

Data are presented as the mean values ± standard deviation (SD) from at least three independent experiments. Student’s *t* tests were used to compare the differences between groups. One-way ANOVA followed by Tukey’s post-hoc tests were used to compare the differences among multiple groups. Two-way ANOVA was used for the immunofluorescence assay. Statistical analyses were performed using GraphPad Prism 5 (GraphPad Software, USA). *p* < 0.05 was considered statistically significant.

## Results

### bLF increases cell proliferation in DP cells

**Fig. 1:**
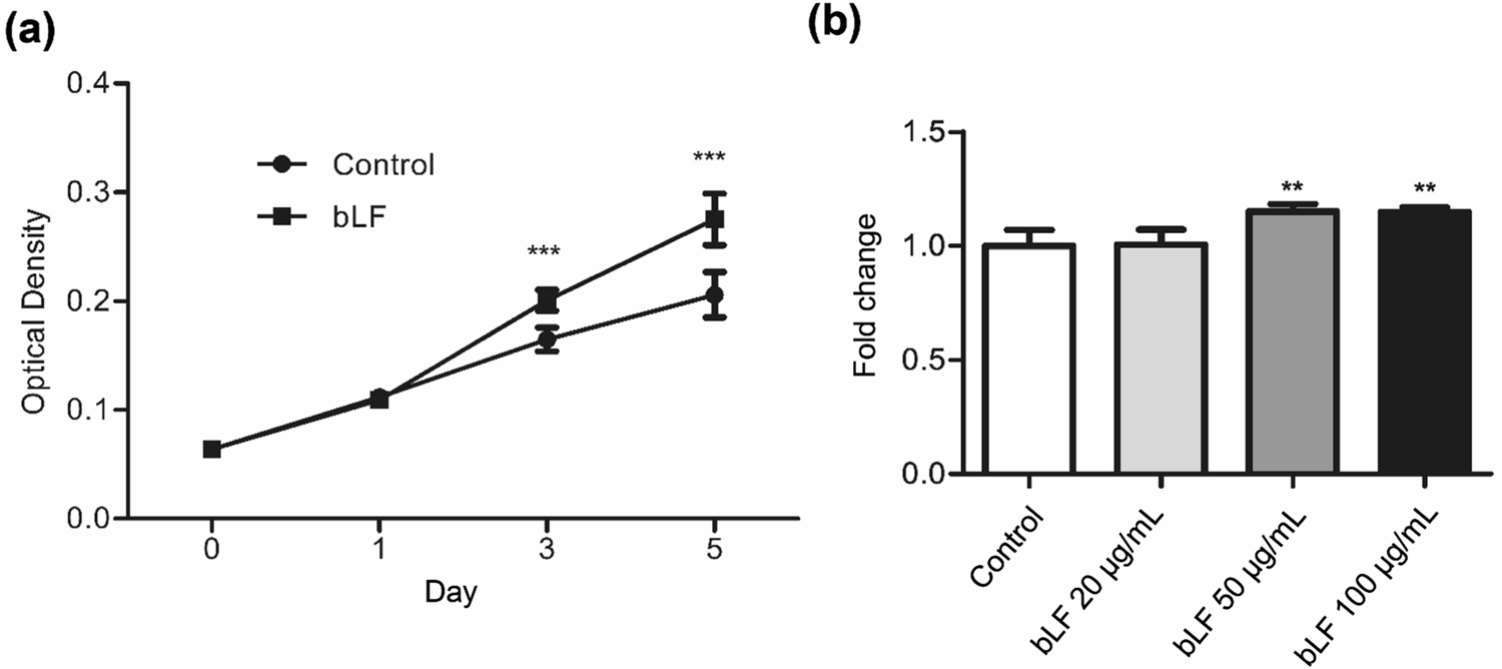
bLF increases cell proliferation in DP cells. **a** DP cells were treated with 50 μg/mL bLF for 0–5 days. **b** Cell growth at different concentrations of bLF at day 5. Cell proliferation was analyzed by the MTT assay. Data are presented as the mean values ± SD from three independent experiments. ***p < 0.001

To investigate whether bLF could regulate cell proliferation, the MTT assay was performed. The results showed that 50 µg/mL of bLF significantly increased the growth rate of DP cells since day 3 (Fig. 1a), and the effect of different concentrations of bLF on DP cells at day 5 is shown (Fig. 1b). According to the results of Fig. 1a, the doubling times of DP cell proliferation for the bLF-treated and control groups were ~ 32 and ~ 37 h, respectively. These findings suggest that bLF increases DP cell proliferation.

### bLF increases phosphorylation of Erk and Akt in DP cells

**Fig. 2:**
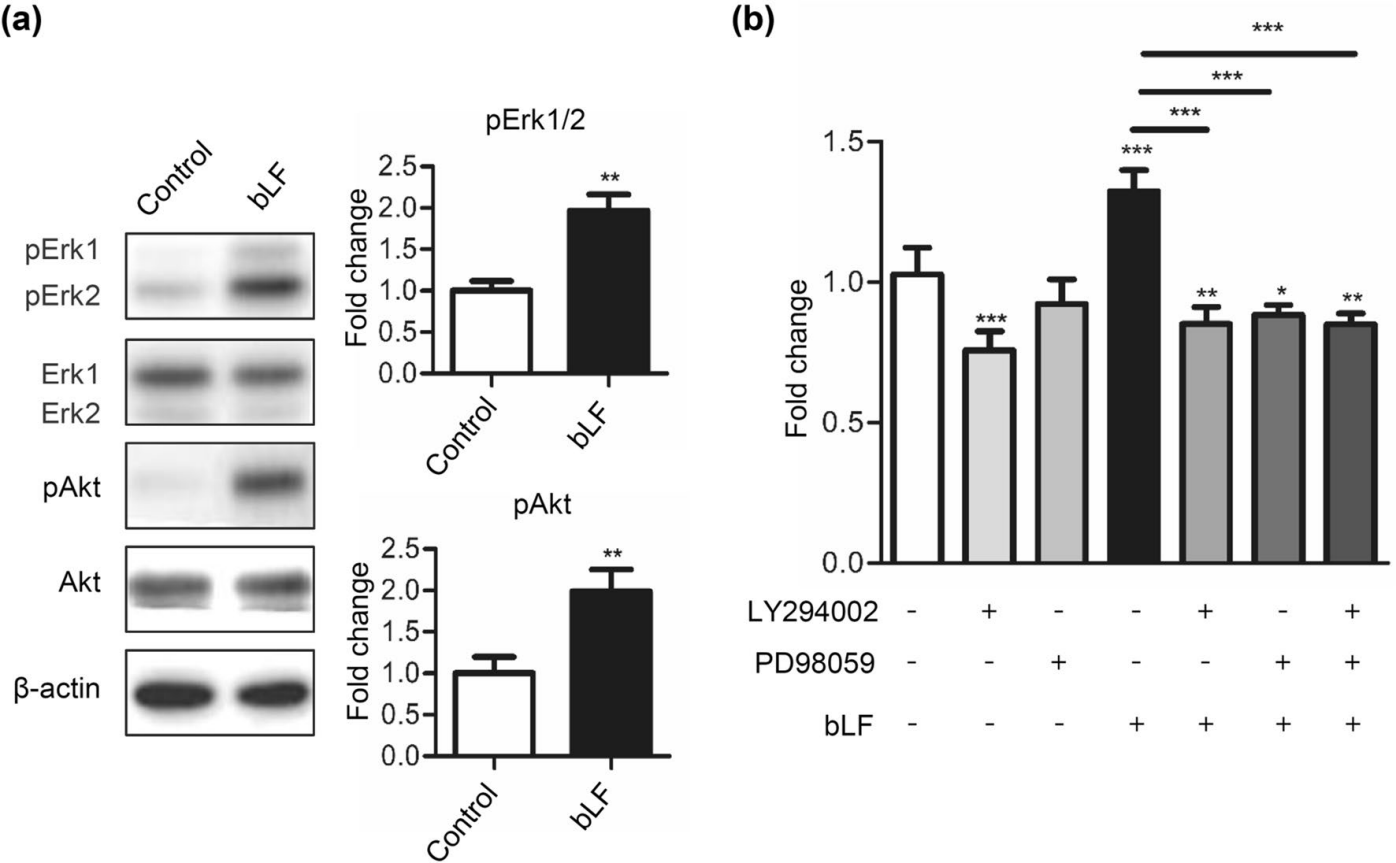
bLF increases phosphorylation of Erk and Akt in DP cells. **a** DP cells were serum-starved for 1 h and subsequently treated with 50 μg/mL of bLF for 1 h. Signals of phosphorylated proteins on Western blot analyses were quantified and normalized to their total protein levels. **b** DP cells treated with 50-μg/mL bLF and 5-μM LY294002, 5-μM PD98059, or a combined treatment of LY294002 and PD 98059 for 3 days. Cell proliferation was analyzed by the MTT assay. Data are presented as the mean values ± SD from three independent experiments. *p < 0.05; **p < 0.01; ***p < 0.001

As it is well known that the Erk and Akt signaling pathways are involved in cell proliferation and differentiation, we analyzed the phosphorylation of Erk and Akt using Western blot analysis. We found that bLF was able to increase the phosphorylation levels of both Erk1/2 and Akt in DP cells (Fig. 2a). To evaluate the role of Akt and Erk activation in bLF-mediated DP cell proliferation, we treated the cells with 5-μΜ LY294002, an Akt phosphorylation inhibitor, and/or 5-μM PD98059, an Erk phosphorylation inhibitor. The MTT assay results showed that PD98059 or LY294002 decreased cell proliferation and also blocked the proliferative effect of bLF at day 3 (Fig. 2b). These findings indicate that bLF promotes cell proliferation through the activation of Erk and Akt signaling in DP cells.

### bLF binds to DP cells

**Fig. 3:**
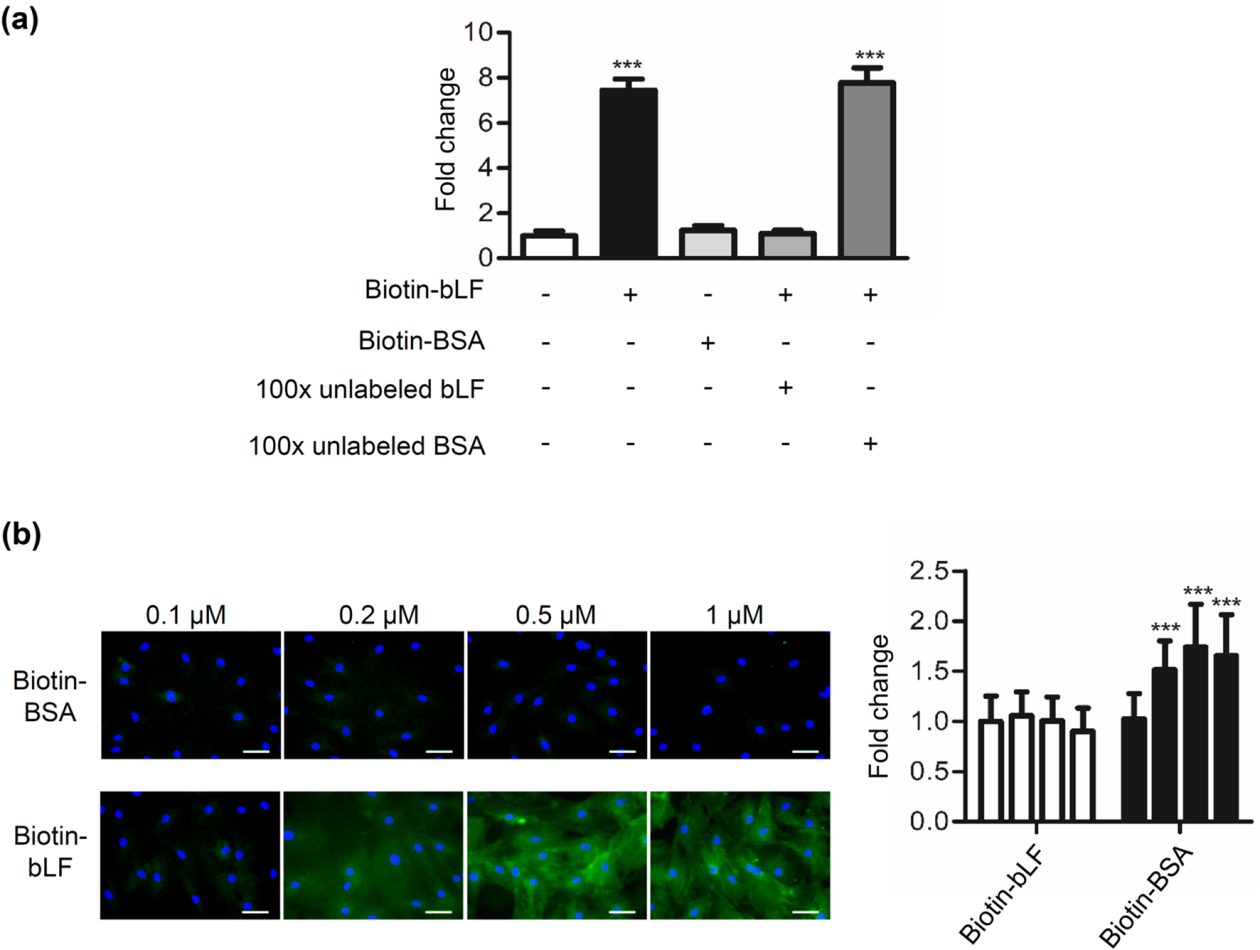
Binding of bLF to DP cells. **a** DP cells were treated with 0.1-μΜ biotin-labeled bLF or biotin-labeled BSA in the presence of 100-fold molar excess of unlabeled bLF or BSA for 4 h at 4 °C. The binding of biotin-labeled bLF to DP cells was detected by incubating them with HRP-conjugated avidin, followed by adding the OPD substrate reagent and measuring the absorbance at 492 nm. **b** Following methanol fixation and permeabilization, cells treated with biotin-labeled bLF or biotin-labeled BSA were stained for streptavidin-FITC Ab (green), and their nuclei were stained with DAPI (blue). Fluorescent microscopy showing DP cells treated with 0.1–1 μΜ of biotin-labeled BSA and biotin-labeled bLF for 1 h. The fluorescent levels of cells bound by biotin-bLF were quantified using AxioVision Software and normalized to the levels of biotin-BSA control. Scale bars, 50 μm. Data are presented as the mean values ± SD from three independent experiments. ***p < 0.001

We examined whether bLF can bind to DP cells. The results showed that biotin-labeled bLF but not biotin-labeled BSA bound to DP cells (Fig. 3a). In the presence of a 100-fold molar excess of unlabeled bLF, the binding was reduced to the control level. By contrast, biotin-labeled bLF binding was not blocked by a 100-fold molar excess BSA. To further investigate and confirm the binding of biotin-labeled bLF was saturated, DP cells were treated with different concentrations of biotin-labeled bLF for 1 h, and the cells were observed under a fluorescence microscope. The results showed that DP cells had strong signals of biotin-labeled bLF binding in the whole cell (Fig. 3b) and the binding of 0.5 μM bLF reached to the plateau. So the saturated concentration of biotin-labeled bLF in DP cells is ~ 0.5 μM. Conversely, biotin-BSA also had relatively weak signals. These findings indicate that bLF specifically binds to DP cells.

### RAP does not compete with bLF for binding to DP cells

**Fig. 4:**
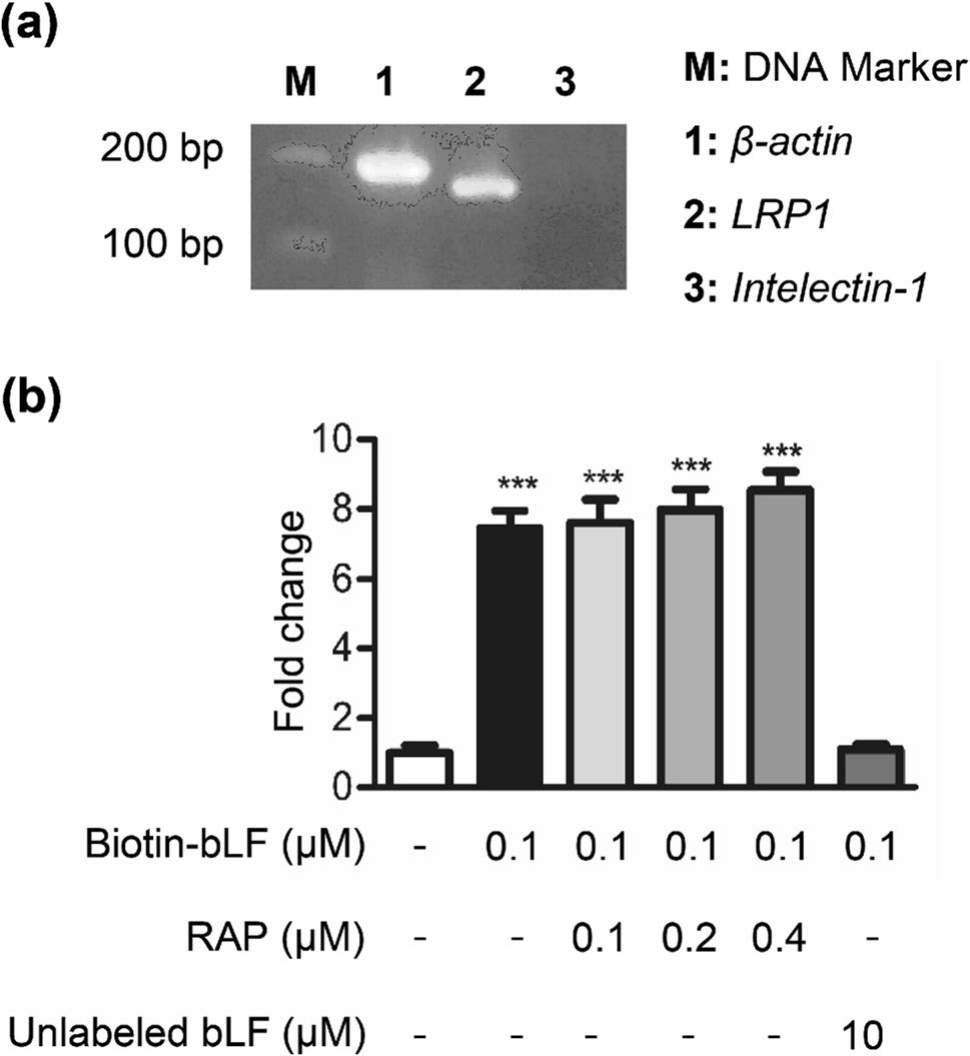
RAP does not compete with bLF for binding to DP cells. **a** The RT-PCR products of *β-actin* (lanes 1), *LRP1* (lanes 2) and *intelectin-1* (lane 3) were separated by an agarose gel. Intelectin-1 is a lactoferrin receptor expressed by the small intestine. **b** DP cells were plated onto 96-well tissue culture plates (1 × 10^4^ cells/well) and incubated with biotin-labeled bLF or biotin-labeled BSA in the presence of the indicated concentrations of RAP (0.1–0.4 μM) for 4 h at 4 °C. The binding of biotin-labeled bLF to DP cells was detected by incubation with HRP-conjugated avidin, which was followed by adding the OPD substrate reagent and measuring the absorbance at 492 nm. Data are presented as the mean values ± SD from three independent experiments. ***p < 0.001

Low-density lipoprotein receptor-related protein 1 (LRP1), one of LF receptors in mammalian cells, is present in human skin keratinocytes and fibroblasts [33]. A previous study indicated that DP cells from wild-type anagen follicles homogeneously express LRP1 in culture [6]. We also examined whether LRP1 can express in rat DP cells. The results of RT-PCR showed that DP cells expressed high levels of *LRP1* mRNA (Fig. 4a). To investigate whether LRP1 is involved in the binding of bLF to DP cells, the cells were treated with biotin-labeled bLF together with RAP. RAP is a high-affinity ligand for LRP used as a universal competitor for LRP ligands [3]. DP cells were incubated with biotin-labeled bLF in the presence of increasing concentrations of RAP (0.1–0.4 μM). However, our data showed that RAP, a protein that efficiently inhibits LF binding to LRP1, did not inhibit biotin-labeled bLF to DP cells (Fig. 4b), suggesting that bLF does not bind to LRP1 on DP cells. It has been reported that intelectin is a LF receptor in the small intestine [31]. Our data showed that intelectin was not expressed in rat DP cells (Fig. 4a). These observations suggest that LRP1 is not involved in the specific binding of bLF to DP cells.

### bLF increases hair growth in C57BL/6 mice

**Fig. 5:**
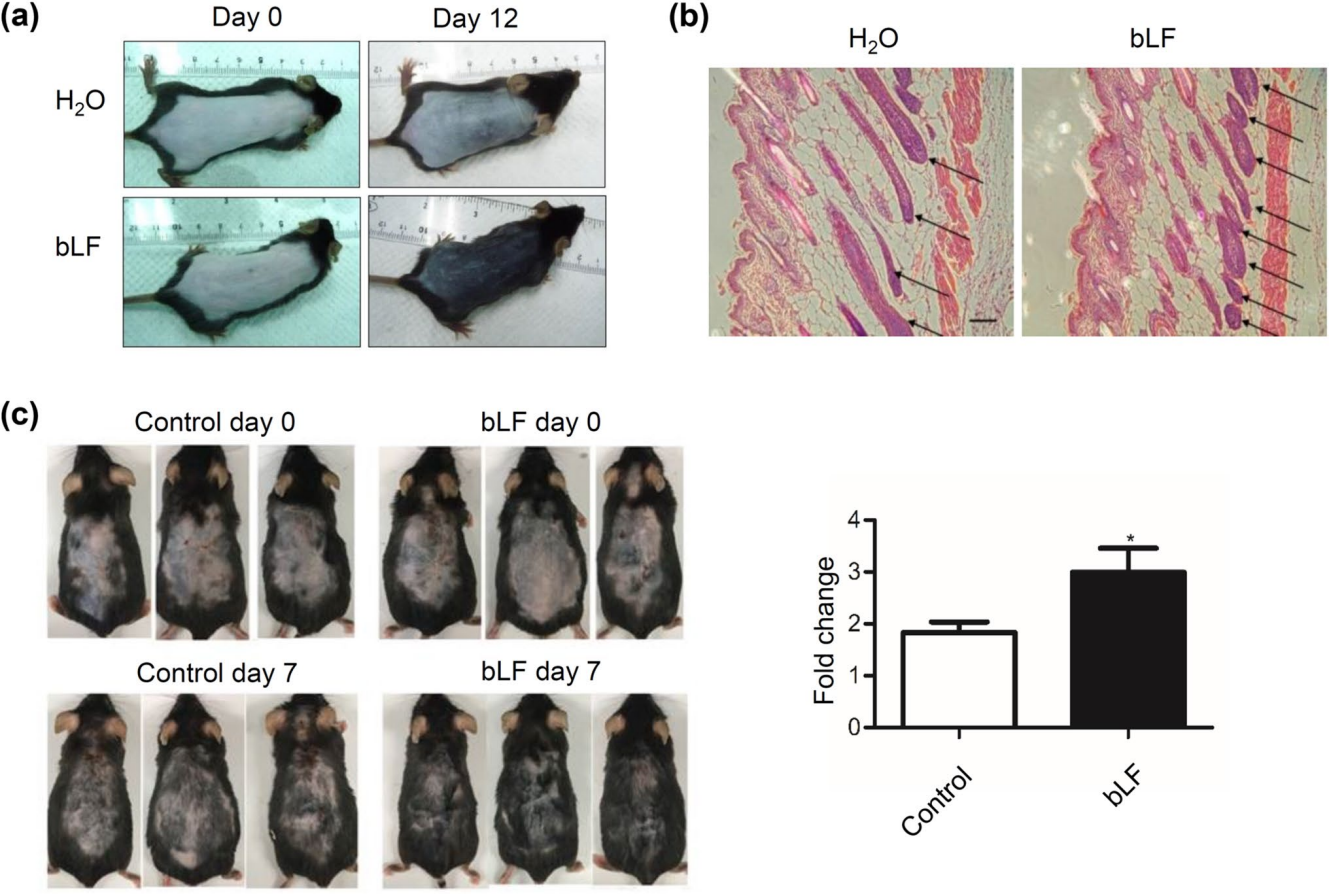
bLF increases hair growth in both young and aged mice. **a** Young mice treated with 200-mg/mL bLF or ddH_2_O. **b** Hemotoxylin and eosin staining of the back skin from the young mice. Longitudinal sections of hair follicles (arrows). Scale bar = 200 μm. **c** Aged mice treated with 40-mg/mL bLF or ddH_2_O control for 7 days. Hair growth was quantified using ImageQuant TL software and normalized to their levels at day 0. *p < 0.05

To assess the effect of bLF on hair loss in vivo, hairs of mice at 2 months and 1 year of age were synchronized by depilation. bLF was applied on the back twice daily for 7–12 days. In young mice, hair growth of the back skin was faster in bLF-treated mice than in control mice (Fig. 5a). Moreover, histological analysis of the back skin showed a significantly increased number of hair follicles in bLF-treated mice (Fig. 5b). In aged mice, we also found that bLF could significantly promote hair growth (Fig. 5c). These findings suggest that bLF increases hair growth in both young and aged C57BL/6 mice.

### bLF increases Wnt signaling-related genes and proteins in DP cells

**Fig. 6:**
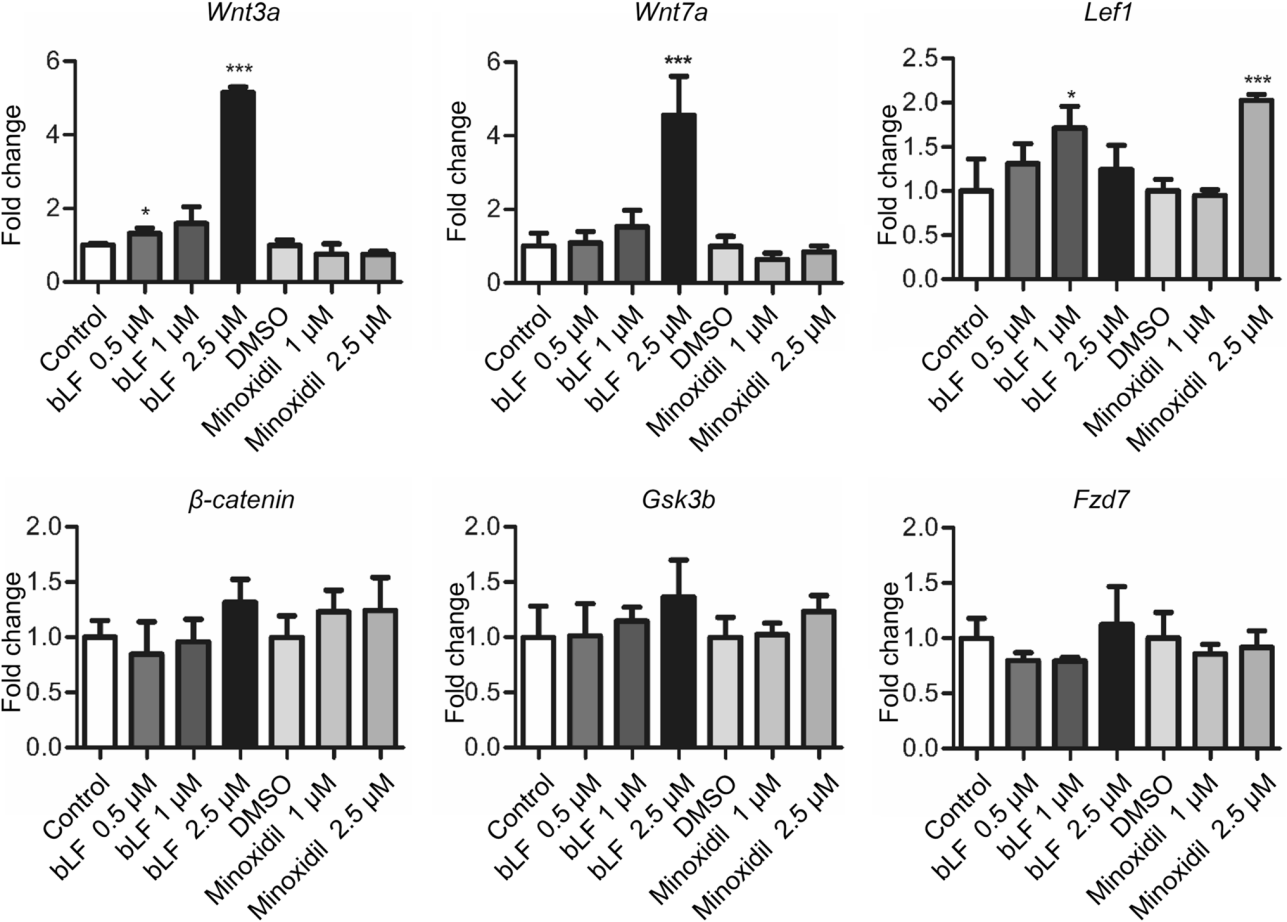
bLF increases the expression of Wnt signaling-related genes in DP cells. The mRNA expression levels of *Wnt3a*, *Wnt7a*, and *Lef1* in DP cells treated with 0.5–2.5 μM of bLF or 1–2.5 μM of minoxidil was analyzed by real-time RT-PCR. Data are presented as the mean values ± SD from three independent experiments. *p < 0.05; ***p < 0.001

**Fig. 7:**
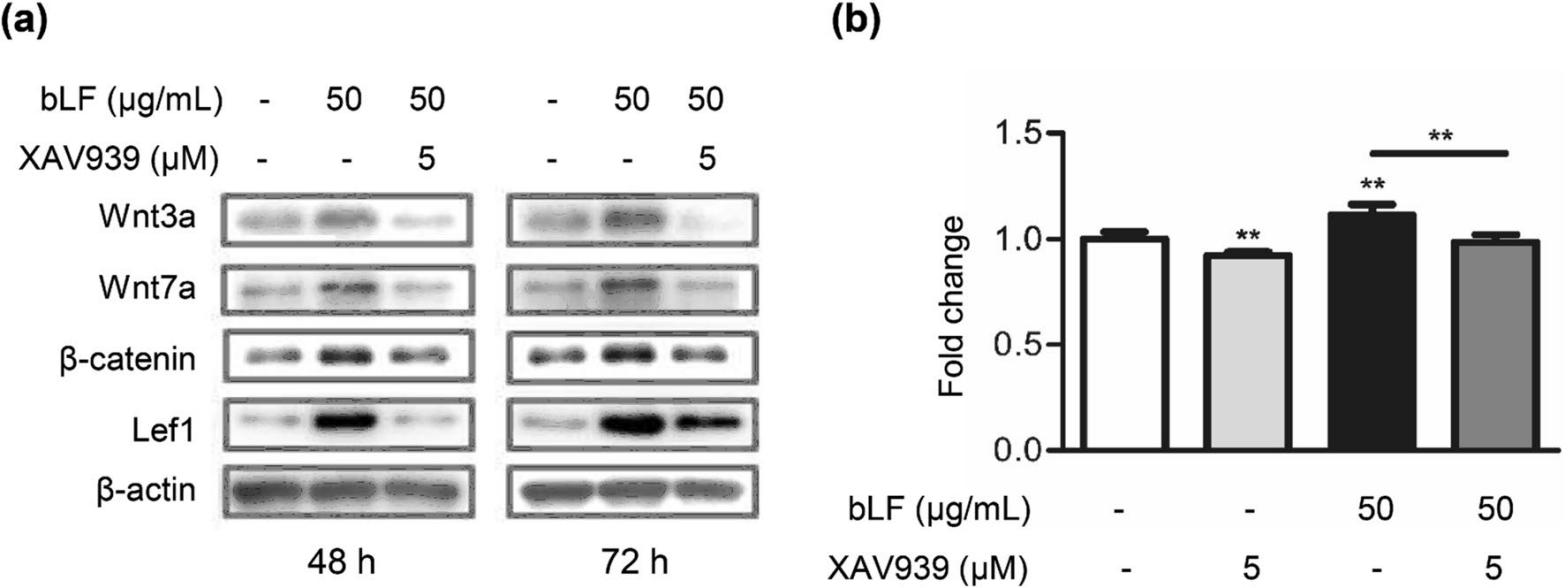
bLF increases the Wnt pathway-related proteins in DP cells. **a** DP cells were treated with 50 μg/mL of bLF and/or 5-μM XAV939 for 48 h (left) or 72 h (right). Signals of proteins on Western blots were quantified and normalized to their total protein levels. **b** Cell viability of DP cells treated with 50-μg/mL bLF and/or 5-μM XAV939 for 5 days. Cell viability was analyzed using the MTT assay. Data are presented as the mean values ± SD from three independent experiments. *p < 0.05; **p < 0.01

The Wnt pathway plays an essential role in hair follicle induction [29]. Therefore, we analyzed whether Wnt signaling-related genes were induced by bLF. DP cells were treated by bLF or minoxidil. Minoxidil is the first and so far the only FDA-approved topical product for the treatment of AGA [37]. The results of real-time RT-PCR indicated that bLF significantly increased the mRNA levels of *Wnt3a*, *Wnt7a*, and *Lef1*, but not of *β-catenin*, *Fzd7*, and *Gsk3b* (Fig. 6). However, minoxidil could only increase the *Lef1* expression. Our results suggest that bLF regulates Wnt signaling pathways which are different from those modulated by minoxidil. We further analyzed the effect of bLF on the expression of the Wnt pathway-related proteins and the effect of the Wnt signaling inhibitor XAV939 on cell proliferation. XAV939 inhibits Wnt signaling by stimulating β-catenin degradation. Western blot analysis showed that bLF increased protein levels of Wnt3a, Wnt7a, β-catenin, and Lef1 for both 48-h and 72-h treatments (Fig. 7a). The bLF-induced increase in cell proliferation could be significantly reversed by XAV939 (Fig. 7b). These results suggest Wnt signaling pathways are involved in bLF-induced proliferation of DP cells.

## Discussion

LF exerts an effect on cell growth and differentiation [23]. In this study, we found that bLF promoted cell proliferation in DP cells and hair growth in mice. bLF increased Erk and Akt signaling independent of LRP1 receptors in DP cells. Moreover, bLF significantly induced the expression of Wnt signaling-related genes. Therefore, we propose that bLF stimulates cell proliferation in DP cells and hair growth in mice through Erk/Akt and Wnt pathways.

DP cell proliferation is important for the morphogenesis and growth of the hair follicles [26]. Erk and Akt signaling pathways are the two major controllers of cell proliferation. The Erk signaling pathway plays key roles in cell proliferation of many cells [39], including DP cells [18, 27]. Akt plays a critical role in mediating survival signals [5]. Moreover, it was reported that the Akt pathway is also involved in regulating the survival of DP cells [9]. Consistently, we found that bLF increases the growth of DP cells and the phosphorylation of Erk and Akt. Moreover, the Erk inhibitor PD98059 and the Akt inhibitor LY294002 significantly blocked bLF-induced proliferative effect in DP cells. Therefore, we propose that bLF is at least partly responsible for promoting the cell proliferation in DP cells by upregulating the Erk and Akt pathways.

LF exerts multiple functions by either binding to the membrane receptors to induce signal transduction pathways or entering the target cells via endocytosis [38]. Human neoplastic cell lines also reportedly have specific LF-binding sites, and a receptor-mediated endocytosis could be supposed [13]. Recent studies suggest that LRP1 functions as both an endocytic receptor and a signaling receptor [11, 19]. LF-induced proliferation and Erk signaling in osteoblastic cells were abrogated by RAP [8], which potently inhibits the binding of ligands to both LRP1 and LRP2 [7]. In our study, bLF can bind to DP cells and can be endocytosed. However, RAP cannot inhibit the binding of bLF to DP cells. These results suggest that LRP1 is not involved in bLF-induced cell proliferation. Other receptors that mediate endocytosis and enhance cell proliferation may exist. Therefore, to understand the complicated process of bLF-induced cell proliferation, it will be of great interest to further investigate the receptor responsible for bLF-mediated cell proliferation.

Hair loss, also known as alopecia, occurs in most people at some time in their life. Thus, it is very important to develop new therapeutic agents to stop hair loss and enhance hair growth. LF is a native and multifunctional glycoprotein in mammal milk which is known to exhibit a wide range of biological activities [22]. According to the previous study, the follicular penetration route may be especially relevant for large molecular weight (MW) molecules [17]. The higher MW dextrans (10-kDa MW) were confined to the follicular structures immediately surrounding the hair shaft [20]. Another paper showed that the FITC-labeled pollen proteins (10–100 kDa) were detected in the dermal tissue around the hair follicle [14]. These results suggest that large molecules could pass through the skin via hair follicles. Therefore, it is likely that lactoferrin can enter the bottom of hair follicles and then induce DP cell proliferation. In this study, the topical use of bLF significantly promoted hair induction in C57BL/6 mice, suggesting that bLF induces hair growth and has great potential for development of anti-alopecia drugs. For clinical applications, native bLF was rapidly degraded with a half-life, in artificial gastric fluid containing pepsin, native bLF was degraded with a half-life of approximately 17 min and plasma half-life of the bLF in rats was 7.8 min [24]. For commercialization of bLF, another paper [15] showed that bLF in the new liquid formulation, containing 1 mg/mL bLf, 1.74 mg/mL arginine, 15% (w/v) trehalose, and 0.02% (v/v) Tween 80 in 100 mM sodium phosphate buffer at pH 6.5, was stable under various storage conditions for 6 months. The formulation for hair-growth promoting products with bLF needs to be further developed.

The Wnt signaling pathway is important for hair morphogenesis and hair cycle [16]. Previous research has found that Wnt proteins play a critical role in normal hair follicle development and cycling [12]. β-Catenin interacts with the members of the Lef/Tcf family of transcription factors to activate gene expression [2]. Our results indicate that bLF significantly increases the mRNA levels of *Wnt3a*, *Wnt7a*, and *Lef1* but not of β-catenin, *Fzd7*, and *Gsk3b*. Consistent with previous findings [16], Wnt3a and Wnt7a can act as inductive signals to maintain the DP in the anagen state. Conversely, our data showed that minoxidil increased the expression of *Lef1* but not of *Wnt3a* and *Wnt7a*. We also found that bLF increased the expression of the Wnt pathway-related proteins, namely Wnt3a, Wnt7a, β-catenin, and Lef1, and that the Wnt pathway inhibitor XAV939 significantly reversed the bLF-induced DP cell proliferation. These results suggest that Wnt signaling pathways are also involved in the bLF-mediated hair growth and that bLF and minoxidil induce hair growth by modulating different factors.

**Fig. 8:**
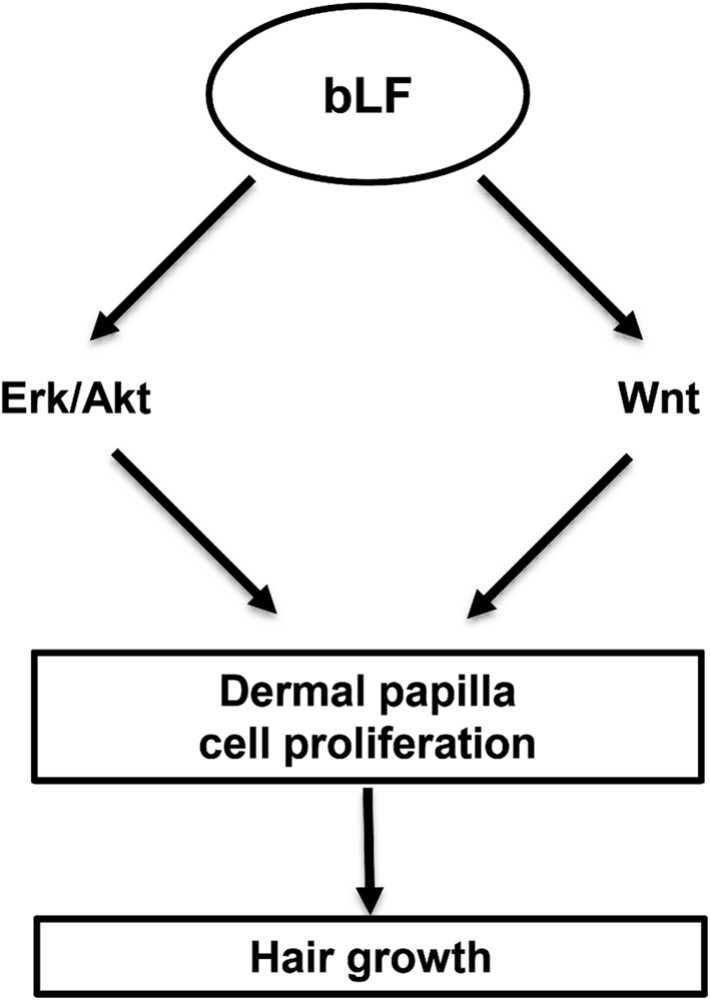
A schematic depicting the potential mechanisms involved in bLF-induced hair growth. *bLF* bovine lactoferrin, *Erk* extracellular signal‐regulated kinase, *Akt* protein kinase B

Taken together (Fig. 8), bLF could induce hair growth in young and aged mice. Mechanistically, bLF stimulates the proliferation of DP cells via Erk/Akt and Wnt signaling pathways, which are independent of LRP1. To our knowledge, we are the first to report bLF as a stimulator of hair growth. Our findings show a great potential of the use of bLF in developing agents to treat alopecia in the future. In the future studies, we will further study the effect of bLF on the stem cell niche or bLF on the proliferation of dermal papilla cells in vivo.
